# Dihydroartemisinin suppresses proliferation, migration, the Wnt/β-catenin pathway and EMT via TNKS in gastric cancer

**DOI:** 10.3892/ol.2021.13152

**Published:** 2021-11-30

**Authors:** Yanmei Ma, Peng Zhang, Qilong Zhang, Xiaofei Wang, Qiong Miao, Xiaolan Lyu, Bo Cui, Honghong Ma

Oncol Lett 22: Article no. 688, 2021; DOI: 10.3892/ol.2021.12949

Following the publication of this article, the authors have reaized that Table II was printed containing certain numerical errors, although these were not significant enough to have affected the statistical results.

The corrected version of Table II is shown on the next page, and the corrected data (concerning the ‘Depth of invasion’, Lymph metastasis’, ‘TNM stage’ and ‘Survival status’ data rows) are highlighted in bold. The authors are grateful to the Editor of *Oncology Letters* for allowing them the opportunity to publish this corrigendum, and all the authors agree to this corrigendum. Furthermore, the authors apologize to the readership for any inconvenience caused.

## Figures and Tables

**Table II.** Association between TNKS expression and the clinicopathological characteristics of patients with gastric cancer (n=87).

		TNKS expression			
Characteristic	Total (n=87)	Positive, % (n=61)	Negative, % (n=26)	χ^2^	P-value
Sex					
Male	42	28 (66.7)	14 (33.3)	0.461	0.497
Female	45	33 (73.3)	12 (26.7)		
Age, years					
<60	43	32 (74.4)	11 (25.6)	0.752	0.386
≥60	44	29 (65.9)	15 (34.1)		
BMI					
<24	27	17 (63.0)	10 (37.0)	0.978	0.613
24-27.9	31	23 (74.2)	8 (25.8)		
≥28	29	21 (72.4)	8 (27.6)		
Smoking					
No	46	33 (71.7)	13 (28.3)	0.123	0.726
Yes	41	28 (68.3)	13 (31.7)		
Drinking					
No	39	24 (61.5)	15 (38.5)	2.481	0.115
Yes	48	37 (77.1)	11 (22.9)		
*Helicobacter pylori* infection					
No	54	37 (68.5)	17 (31.5)	0.173	0.677
Yes	33	24 (72.7)	9 (27.3)		
Depth of invasion					
T1/T2	**39**	**22 (56.4)**	17 **(43.6)**	**6.336**	0.012^a^
T3/T4	**48**	**39 (81.2)**	**9 (18.8)**		
Lymph metastasis					
N0	**26**	**12 (46.2)**	**14 (53.8)**	**10.160**	0.001^b^
N1/N2/N3	**61**	**49 (80.3)**	**12 (19.7)**		
TNM stage					
Ⅰ-II	40	**23 (57.5)**	**17 (42.5)**	**5.623**	0.018^a^
III-IV	47	**38 (80.9)**	**9 (19.1)**		
Survival status					
Dead	33	**31 (93.9)**	**2 (6.1)**	**14.402**	<0.001^c^
Alive	54	**30 (55.6**)	**24 (44.4)**		

a P<0.05;

b P<0.01;

c P<0.001. TNKS, Tankyrases; TNM, tumor-node-metastasis.

